# Etiology and duration of the disease in the assessment of intellectual functioning of pediatric patients with epilepsy: An observational study

**DOI:** 10.1016/j.heliyon.2023.e14085

**Published:** 2023-02-25

**Authors:** Viola Oldrati, Sara Minghetti, Nicoletta Zanotta, Alessandra Bardoni, Claudio Zucca

**Affiliations:** Scientific Institute IRCCS Eugenio Medea, 23842 Bosisio Parini, Lecco, Italy

**Keywords:** Pediatric epilepsy, Global intellectual functioning, Duration of epilepsy, Etiology

## Abstract

Childhood epilepsy can be frequently associated with impaired cognitive functioning. Previous research has suggested an increased risk of cognitive impairment that may be related to the etiology, the electro-clinical pattern and the load of anti-seizure medications (ASMs). The aim of this study was to evaluate the impact of different clinical features on the global intellectual functioning in a cohort of children and adolescents with epilepsy. We studied eighty patients diagnosed and followed in a tertiary care center. These factors were examined: 1. Etiology of epileptic syndrome; 2. Type of seizure; 3. Number of ASMs; 4. Seizure frequency; 5. Age at seizure onset; 6. Total duration of epilepsy; and 7. Active duration of epilepsy. Multiple regression analysis showed that the etiology and the total duration of epilepsy were the best indicators of intellectual functioning. The present data indicate that children with symptomatic epilepsy (SE) have lower IQ scores (M = 63.5), while children with self-limited focal epilepsy and generalized idiopathic epilepsy, i.e. age-related epileptic syndromes (ARES), have a higher IQ (M = 100.0; p < 0.01). Children with epilepsy of unknown etiology (UEE) (M = 75.1; p < 0.05) are positioned at an intermediate level between the SE and the ARES group (p < 0.01). Increased duration of epilepsy was associated with decreased intellectual functioning. In conclusion, knowledge about the risks associated with etiologic factors and the duration of the disease may guide the definition of optimal neuropsychological rehabilitation strategies.

## Highlights

• Etiology of epilepsy and the total duration of the disease were the best indicators of global intellectual functioning.
• Increased total duration of epilepsy was associated to lower scores in all cognitive indexes.
• Knowledge about the risks of etiology-related factors may help planning neuropsychological rehabilitation strategies.

## 1 Introduction

Children and adolescents with epilepsy may show some degree of cognitive impairment [1,2]. Indeed, a recent cohort study estimated that 2% of children with a diagnosis of epilepsy in the first 10 years of life were identified as having cognitive impairment and that the prevalence of cognitive impairment was higher of than in controls in all the categories of severity considered [3].

A substantial number of studies trying to identify the predictors of cognitive development in this clinical population have largely focused on seizure-related factors. Indeed, refractory epilepsy [4], seizures' frequency [5,6], duration of the disease [7] and seizures' early onset [8,9] have been associated with cognitive deficits. Patients with generalized seizures have worse cognitive performance than those with focal seizures [10,11].

Nevertheless, the variability of the diagnostic criteria and clinical and therapeutical management of the patients may affect the impact of these variables on cognitive outcomes (see Seidenberg et al., 2007 [12] and Menlove and Reilly 2015 [13] for reviews).

Furthermore, there is more and more evidence, mainly from genetic studies, that physiological and molecular causes of epileptic syndromes are responsible not only for seizures, but also for functional abnormalities leading to cognitive and behavioral impairment [14]. A study found that about 25% of a sample of children newly diagnosed with the once called idiopathic epilepsies needed special education services even before seizure onset [15]. In various genetic etiologies causing infantile epileptic syndromes, the degree of intellectual disability can be severe even with a satisfying response to the anti-seizure medications (ASMs) [2,16].

These data suggest that both epilepsy and cognitive impairment may originate from a common underlying pathology [17], rather than one causing the other. Given that epileptic syndromes have heterogeneous etiologies and distinct pathogenic mechanisms, this may contribute to the variability observed in the cognitive development of children with epilepsy [18]. This also justifies why, even among patients who show difficulties, different trajectories of development can be found [19]. This view stresses the need to consider the mutual link among etiology, electro-clinical picture and intellectual functioning in order to understand the complex relationship between cognition and epilepsy [20]. It follows that the definition of distinct diagnostic groups based on the etiology of the disease and of the related risk factors remains a non-negligible issue to consider to understand the cognitive profiles that can be uncovered in childhood epilepsy.

The aim of the present observational study was to examine the impact of clinical features, including seizure type and etiology, on the intellectual functioning in a cohort of children and adolescents with epilepsy. In addition to these factors, our analysis focused on age at onset and duration of the epileptic syndrome, seizure frequency and number of ASMs.

## 2 Methods

### 2.1 Study design

This study included a cohort of children and adolescents with a diagnosis of epilepsy, followed at a neuro-pediatric rehabilitation center in Italy (Scientific Institute I.R.C.C.S. E. Medea, Bosisio Parini, Italy). Patients diagnosed by the Epilepsy Center of the Institute can be referred to the Neuropsychology Unit to assess the level of intellectual functioning and, whenever considered necessary, set up a rehabilitation program. In these cases, the level of intellectual functioning is determined, as part of routine diagnostic assessment, by means of standard batteries (i.e., the age-appropriate Wechsler Intelligence Scale tests). Moreover, additional in-more-depth investigations of the neuropsychological profile are custom-tailored to each patient.

### 2.2 Participants and clinical features selection

Patients were included in this study if they were: (i) diagnosed with epilepsy, (ii) aged between 5 and 18 years at assessment; (iii) mentally and physically able to undergo a cognitive assessment. The history of a past medical neurosurgical intervention and/or the diagnosis of neurological progressive disorders constituted exclusion criteria, in order to examine the “natural course” of the disease without other cofounding factors. Classification of type and etiology of the epileptic syndromes were determined by two neurologists (CZ, NZ) and a child neurologist (SM) according to the ILAE classifications [[21], [22], [23]]. The correct classification was reviewed based on the familial and clinical history and on the detailed analysis of diagnostic data, particularly the electroencephalograms (EEG) and neuroimaging.

Patients were distinguished into the following diagnostic categories: (i) self-limited focal and generalized idiopathic epilepsies, that is patients with age-related epileptic syndromes (ARES) such as Self-limited Epilepsies with Centro-temporal Spikes or Childhood Absence Epilepsies; (ii) focal or generalized or combined focal and generalized epilepsies with suspected but unknown etiology (UEE) i.e. cryptogenic epilepsies, and lastly (iii) focal or generalized or combined focal and generalized epilepsies with known etiology i.e. symptomatic epilepsy (SE). The ASM variable indicates whether the participants took (i) no medication (no therapy); (ii) only one medication (monotherapy); or (iii) two or more medications (polytherapy). For what concerns the seizure frequency, participants were classified as follows: (i) seizure free, if no seizure was notified for at least 1 year; (ii) low frequency, ranging from one seizure in the previous year up to less than one seizure per month; and (iii) high seizure frequency, ranging from one seizure per month to daily seizure. Finally, alongside age at seizure onset, the duration of epilepsy was collected, reflecting either (i) the time (expressed in months) passed from the first notified seizure to the cognitive assessment, i.e., the total duration of epilepsy, or (ii) the time (expressed in months) passed from the first to the last notified seizure, i.e., the active duration of epilepsy, as computed in Lopes et al. [24]. In patients with non-controlled seizures, the total duration equals the active duration of the disease, whereas in those with controlled seizures, the total duration is longer than the active duration of the disease. For most patients, both the EEG recording, conducted for either diagnostic or monitoring purpose, and the cognitive assessment took part within a few days/weeks, but extended to months in some cases.

**Fig. 1:**
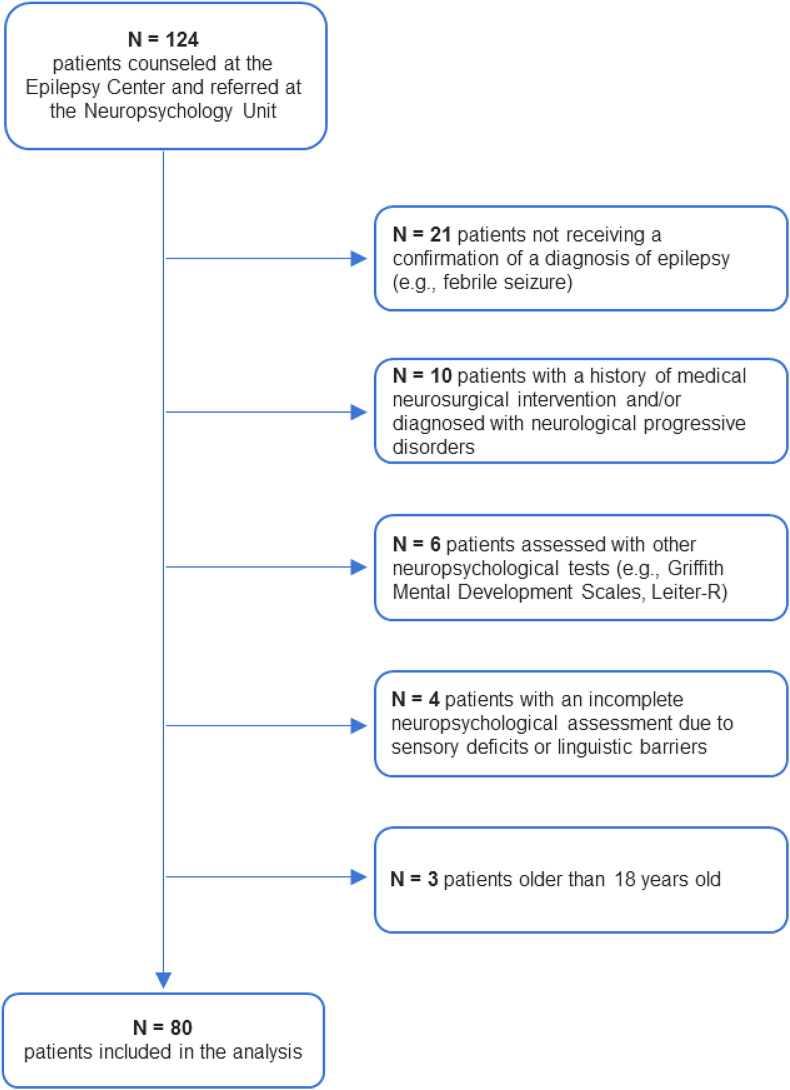
Flowchart of patients' selection for inclusion in the analysis.

Demographic and clinical data and diagnostic exams were retrieved from patient medical records. Approval was received from the local ethical standards committee on human experimentation at the Scientific Institute I.R.C.C.S. E. Medea. The study was conducted in agreement with the Declaration of Helsinki. Due to the observational nature of the study, the local Ethics Committee only required notification about the study (identification number: 13.2021 Oss; date of approval: 24 November 2021). Therefore, written informed consent was not required from the parents/caregivers prior to study enrollment. Fig. 1 depicts the procedure for the selection of patients to be included in the analysis.

### 2.3 Participants

The current study was based on a cohort of 80 children and adolescents between 5 and 18 years of age at assessment, diagnosed with epilepsy of different etiologies. Most patients (77.5%) were taking at least one ASMs at the time of neuropsychological testing. Thirty-two patients (40%) were diagnosed with intellectual disability as defined by an IQ score <70.

### 2.4 Cognitive outcome measures

For each patient, the Full-Scale Intelligence Quotient (FSIQ), the Verbal IQ (VIQ) and the Performance IQ (PIQ) - derived from the age-corresponding Wechsler Intelligence Scale (Wechsler Intelligence Scale for Children, Third or Fourth Edition: WISC III or IV [25,26]; Wechsler Preschool and Primary Scale of Intelligence, Third Edition: WPPSI-III [27]) - were obtained. The FSIQ is considered the most representative indicator of general intellectual ability. The VIQ score is a measure of acquired knowledge, verbal reasoning, and attention to verbal materials, whereas the PIQ reflects abilities such as fluid reasoning, spatial processing and visual-motor integration. As in the routine clinical practice, the neuropsychological evaluation and test scoring were carried out by trained psychologists.

### 2.5 Data diagnostic and statistical analysis

Separate multiple linear regression models were computed on the whole sample to estimate each Wechsler Intelligence Scale quotients (i.e., FSIQ, VIQ and PIQ) from a set of six explanatory variables: 1. Etiology of epilepsy (ARES vs. UEE vs. SE); 2. Type of seizure (focal vs. generalized; focal seizure with secondary generalization were classified as focal); 3. ASMs (no therapy vs. monotherapy vs. polytherapy); 4. Seizure frequency at the time of assessment (seizure free vs. low frequency vs. high frequency); 5. Age at seizure onset (in months); and 6. Total duration of epilepsy (in months) or 7. Active duration of epilepsy (in months). In each model, the main effects were tested. Total duration and active duration of epilepsy were entered in the models separately in order to avoid multicollinearity problems, being the two variables highly correlated (r = 0.76, p < 0.0001). Thus, a total of six multiple linear regression models were computed.

A sample of 80 participants was considered sufficient to estimate models with six explanatory variables according to the rule of thumb, suggesting to include 10/15 observations per number of predictors [28]. Interaction effects were not investigated due to the unequal and small number of observations within the levels of the selected factors (see Table 2).

**Table 1:** Number (n) and percentage (%) or mean (M) and standard deviation (SD) of clinical variables of the sample (k = 80) used for statistical analysis. ABI = acquired brain injury, including perinatal asphyxia or infections, stroke, traumatic brain injury and brain tumor.

	n (%)
sex	
M	40 (50.0)
F	40 (50.0)
**etiology**	
age-related epileptic syndromes	15 (18.8)
unknown etiology	39 (48.8)
symptomatic	26 (32.5)
*ABI*	7 (26.9)
*genetic*	12 (46.1)
*malformations of cortical development* *bilateral white matter lesions* *diffuse supra-tentorial atrophy*	5 (19.2) 1 (3.9) 1 (3.9)
**seizure type**	
focal	54 (67.5)
generalized	26 (32.5)
**ASMs**	
no therapy	18 (22.5)
monotherapy	40 (50.0)
polytherapy	22 (27.5)
**seizure frequency**	
seizure free	45 (56.3)
low frequency	13 (16.3)
high frequency	22 (27.5) **M (SD)**
age at onset (month)	64.8 (44.9)
age at assessment (month)	129.8 (39.8)
total duration of epilepsy (month)	64.6 (44.6)
active duration of epilepsy (month)	44.9 (43.9)
Full-scale IQ	76 (24.0)
Verbal IQ	84 (21.6)
Performance IQ	83 (22.5)

**Table 2:** Number (n) and percentage (%) or mean (M) and standard deviation (SD) of clinical variables according to etiology and comparisons between etiology groups (by Chi-square or Kruskal-Wallis tests). * indicates p < 0.05; ** indicates p < 0.01.

	n (%)			χ (df)
	ARES	UEE	SE	
**sex**				
M	7 (46.7)	19 (48.7)	12 (46.2)	
F	8 (53.3)	20 (51.3)	14 (53.8)	
**seizure type**				
focal	8 (53.3)	26 (66.7)	20 (76.9)	*2.5 (2)*
generalized	7 (46.7)	13 (33.3)	6 (23.1)	
**ASMs**				
no therapy	4 (26.7)	13 (33.3)	1 (3.8)	16.6 (4)**
monotherapy	8 (53.3)	21 (53.8)	11 (42.3)	
polytherapy	3 (20.0)	5 (12.8)	14 (53.8)	
**seizure frequency**				
seizure free	9 (60.0)	25 (64.1)	11 (42.3)	*7.8 (4)*
low frequency	4 (26.7)	6 (15.4)	3 (11.5)	
high frequency	2 (13.3)	8 (20.5)	12 (46.2)	
	**M (SD)**			**H(df)**
		
age at onset (month)	82.1 (35.5)	58.4 (42.8)	64.5 (51.5)	*3.8 (2)*
age at assessment (month)	118.1 (26.3)	128.9 (42.7)	137.9 (41.2)	*2.3 (2)*
total duration (month)active duration (month)	35.6 (28.3) 17.4 (21.6)	70.2 (45.5) 44.3 (42.5)	73.0 (45.5) 61.8 (48.3)	8.9 (2)* 12.9 (2)**

Additionally, diagnostic tests were conducted on the models to check that regression assumptions were met. In more detail, the absence of heteroscedasticity of residuals was tested by means of the NCV test (all models: p > 0.2) and the residual autocorrelation was tested by means of the Durbin-Watson test (all models: p > 0.5). Lastly, a variation inflation factor (VIF) test was performed to check for the multicollinearity assumption (all models: for all the included factors VIF < 1.9).

The level of statistical significance in all tests was defined as p < 0.05. F-statistics and statistical significance was obtained by a Type III SS method. Dunn tests (with Holm-Sidak adjustment for multiple comparisons) were performed to further explore statistically significant main effects. R software (version 4.0.3; R Foundation for Statistical Computing) was used to perform all the statistical analysis.

## 3 Results

Descriptive statistics were performed to provide a depiction of the clinical characteristics of the patients (Table 1).

Table 2 reports the clinical characteristics of patients according to the etiology of epilepsy – i.e., UEE and SE – and the comparison among groups with regards to the factors included in the main statistical analyses. For this purpose, Chi-square test was used to test for frequency differences in categorical variables, whereas the Kruskal-Wallis test was applied for continuous variables. If the Kruskal-Wallis test yielded a significant result, the Dunn test for unequal sample sizes (with Holm-Sidak adjustment for multiple comparisons) was applied to determine which groups differ to each other.

As shown in Table 2, the ASM factor distinguished the clinically classified groups. Both total and active duration of epilepsy differed among groups with statistical significance. The Dunn test showed that the epilepsy of the children with SE had a longer total duration when compared to the children with ARES (p = 0.01) but not to children with UEE (p = 0.42) and that children with UEE had a longer duration of epilepsy compared to children with ARES (p = 0.01). For what concerns the active duration of the disease, the analysis showed that children with SE had a longer duration when compared to the children with ARES (p = 0.01) and children with UEE (p = 0.05), and that children with UEE had a longer duration of epilepsy compared to children with ARES (p = 0.01).

**Fig. 2:**
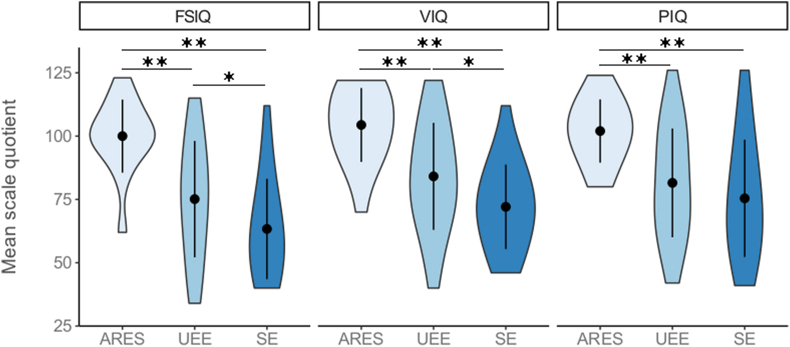
Violin plots depicting the frequency of observations of Weschler quotients (FSIQ, VIQ and PIQ) by Kernel density estimation among groups (ARES vs. UEE vs. SE). The black dots represent mean scores and the error bars represent ±1 SD. * indicates p < 0.05; ** indicates p < 0.01.

Regarding the main aim of the study, separate multiple linear regression models were computed to estimate each Wechsler Intelligence Scale quotients from the set of explanatory factors. The first three models reported in this section (Model 1,2 and 3) included the total duration of epilepsy. First, the model on FSIQ was statistically significant (Adj-R^2^ = 0.39, F (9,70) = 6.8, p < 0.0001). The analysis showed that etiology predicted the FSIQ (F (2) = 9.7, p < 0.001). The Dunn test indicated that all groups differed from each other, with the SE group being the one reporting the lowest FSIQ (M = 63.5, SEM = 3.9), the ARES group the highest (M = 100.0, SEM = 3.7) and the UEE group collocating between the other two groups (M = 75.1, SEM = 3.7) (Fig. 2). The effect of seizure type fell short of statistical significance (p = 0.07), as indicated by lower FSIQ in participants with generalized seizure (M = 73.2, SEM = 4.8) as compared to those with focal seizure (M = 77.2, SEM = 3.6). Moreover, it was found that epilepsy duration significantly predicted FSIQ score (F (1) = 12.8, p < 0.001). Indeed, the regression coefficient (β = −0.43) indicates that the longer the duration of epilepsy the lower the FSIQ. No other factors significantly predicted FSIQ score (all p > 0.3).

The model on Verbal IQ was statistically significant (Adj-R^2^ = 0.37, F (9,70) = 6.1, p < 0.0001). The factor etiology predicted the VIQ score (F (2) = 9.2, p < 0.001). The results of the Dunn test confirmed what emerged in relation to the FSIQ, with the SE group reporting the lowest Verbal IQ (M = 72.1, SEM = 3.3), the ARES group the highest (M = 104.4, SEM = 3.8) and the UEE group collocating between the two (M = 84.1, SEM = 3.4) (Fig. 2). It was found that epilepsy duration significantly predicted the VIQ (F (1) = 14.5, p < 0.001). As observed in the previous model, the regression coefficient (β = −0.47) indicates that the longer the duration of epilepsy the lower the VIQ. No other factors significantly predicted the VIQ (all p > 0.2).

Lastly, the model on PIQ was statistically significant (Adj-R^2^ = 0.22, F (9,70) = 3.4, p < 0.01). The etiology was again a significant factor (F (2) = 4.0, p < 0.05). The Dunn test showed a significant difference between the ARES group (M = 102.0, SEM = 3.2) and both the UEE group (M = 81.5, SEM = 3.4) and the SE group (M = 75.4, SEM = 4.6), but not between these last two (see Fig. 2). Furthermore, the model showed that epilepsy duration significantly predicted PIQ score (F (1) = 5.4, p < 0.05), with a regression coefficient β = −0.32, indicating that the longer the duration of epilepsy the lower the PIQ. No other factors significantly predicted PIQ (all p > 0.3). Table 3 reports regression parameters for models including the total duration of epilepsy.

**Table 3:** Standardized beta coefficients (β), standard errors (SE) and related p-values of the explanatory factors by model including the total duration of epilepsy.

		β (SE)	p-value
Model 1 - FSIQ			
	age at onset	−0.13 (0.1)	0.25
	(total) epilepsy duration	−0.43 (0.1)	<0.001
	etiology (ARES)	0.19 (5.4)	0.08
	type (focal)	−0.18 (5.1)	0.07
	ASMs (no therapy)	0.18 (6.8)	0.14
	seizure frequency (seizure free)	0.02 (7.2)	0.91
Model 2 -Verbal IQ			
	age at onset	−0.23 (0.1)	0.07
	(total) epilepsy duration	−0.47 (0.1)	<0.001
	etiology (ARES)	0.25 (4.9)	<0.05
	type (focal)	−0.12 (4.7)	0.24
	ASMs treatment (no therapy)	0.13 (6.2)	0.29
	seizure frequency (seizure free)	0.05 (6.6)	0.65
Model 3 - Performance IQ			
	age at onset	−0.03 (0.1)	0.85
	(total) epilepsy duration	−0.32 (0.1)	<0.05
	etiology (ARES)	0.08 (5.7)	<0.05
	type (focal)	−0.13 (5.5)	0.26
	ASMs (no therapy)	0.15 (7.2)	0.26
	seizure frequency (seizure free)	−0.01 (7.7)	0.93

The following three models included the active duration of epilepsy (Model 4,5, and 6). The model on FSIQ was statistically significant (Adj-R^2^ = 0.32, F (9,70) = 5.0, p < 0.0001). The analysis showed that etiology predicted the FSIQ (F (2) = 9.8, p < 0.001). No other factors significantly predicted FSIQ (all p > 0.1). The model on Verbal IQ was statistically significant (Adj-R^2^ = 0.34, F (9,70) = 6.1, p < 0.001). The factor etiology predicted VIQ score (F (2) = 9.4, p < 0.001). No other factors were found to be significant predictors (all p > 0.2). Lastly, the model on PIQ was statistically significant (Adj-R^2^ = 0.28, F (9,70) = 3.1, p < 0.01). The etiology was again a significant factor (F (2) = 4.2, p < 0.05). No other factors significantly predicted PIQ (all p > 0.1). Table 4 reports regression parameters for models including the active duration of epilepsy.

**Table 4:** Standardized beta coefficients (β), standard errors (SE) and related p-values of the explanatory factors by model including the active duration of epilepsy.

		β (SE)	p-value
Model 4 - FSIQ			
	age at onset	−0.02 (0.1)	0.89
	(active) epilepsy duration	−0.20 (0.1)	0.09
	etiology (ARES)	0.19 (5.6)	0.11
	type (focal)	−0.12 (5.4)	0.26
	ASMs (no therapy)	0.16 (7.2)	0.20
	seizure frequency (seizure free)	−0.04 (7.6)	0.76
Model 5 -Verbal IQ			
	age at onset	−0.02 (0.1)	0.84
	(active) epilepsy duration	−0.15 (0.1)	0.22
	etiology (ARES)	0.25 (5.4)	<0.05
	type (focal)	−0.05 (5.0)	0.66
	ASMs (no therapy)	0.12 (6.8)	0.38
	seizure frequency (seizure free)	0.03 (7.1)	0.85
Model 6 - Performance IQ			
	age at onset	−0.09 (0.1)	0.42
	(active) epilepsy duration	−0.23 (0.1)	0.09
	etiology (ARES)	0.08 (5.9)	0.61
	type (focal)	−0.09 (5.5)	0.44
	ASMs (no therapy)	0.14 (7.3)	0.31
	seizure frequency (seizure free)	−0.04 (7.8)	0.81

Explorative analyses were carried out to examine potential differences in the intellectual functioning measures between the diagnostic sub-groups within the SE group (i.e., ABI vs. genetic epilepsy vs. other; see Table 1 for further details on the latter sub-group). A series of Kruskal-Wallis tests yielded non-significant results (FSIQ: H (2) = 0.1, p = 0.96; VIQ: H (2) = 0.7, p = 0.72; PIQ: H (2) = 0.1, p = 0.95), indicating that three diagnostic sub-groups did not differ in the considered intellectual functioning measures.

## 4 Discussion

The present findings suggest that, in our cohort of 80 patients, the etiology of epilepsy and the total duration of the disease were the best indicators of global intellectual functioning. In more detail, our data indicate that children with SE had lower FSIQ and VIQ scores than children with ARES and UEE, as well as lower PIQ scores than children with ARES but not UEE. Similar results have been obtained in previous studies showing that children with SE had a statistically low level of global intelligence as compared to patients with other epilepsy syndromes, including ARES [29]. Accordingly, educational underachievement has been observed as prominent for symptomatic epilepsies, pointing to a dominant impact of the underlying etiology on the cognitive outcome, linked to brain dysfunctions or damages [30]. The graphic visualization of the Weschler quotients (see Results, Fig. 2) show that, in our sample, the UEE and SE groups display fairly similar distributions, reflecting a range of scores varying from very low to above the normative level. On the contrary, the ARES group has a less scattered distribution, with most observations settling around the normative level for all the three indexes. Interestingly, a study whose aim was to validate a separate classification of UEE by discriminant function analysis, concluded that this group remains rather difficult to classify, as over half of the children falling within this diagnostic group were once classified as idiopathic (which matches with the classification of ARES used in the present study) or SE [31]. Although the abovementioned study classified different groups of epilepsy according to the amount of EEG interictal epileptiform abnormalities, but not the FSIQ examining patients with an intellectual functioning within the lower border of the normative interval. In our opinion, the inclusion of patients with intellectual disabilities, as in our study, may have enhanced the differences in IQ among the three groups. The fact that the UEE group displays a wide distribution of intelligence scores supports the idea that this group actually consists of patients that are probably “presumed symptomatic”. Possibly, the development of diagnostic techniques, especially in the genetic and neuroimaging fields, will allow to include these patients in the group of symptomatic epilepsies, as already suggested [31,32]. Also the ARES group may be considered, to some extent, “presumed symptomatic”, as it is recognized to underlie a polygenic background, although the substantial absence of a family history of epilepsy for these patients may be explained by either a de novo mutation or complex inheritance [22]. The present findings are in line with previous research showing normal intellectual abilities in this group [33]. Accordingly, children with ARES were reported to have higher IQ scores and higher probability of mainstream schooling than those described as cryptogenic and symptomatic [34]. As shown in Fig. 1, only one patient in this group obtained a FSIQ score falling under 80, score indicating below average cognitive ability. Moreover, 13 out 15 patients with ARES (86.7%) were referred to an additional detailed neuropsychological assessment focusing on executive functions and academic competences, suggesting the presence of certain degree of neuropsychological impairment. Our results support that the use of terms “benign” epilepsy is nowadays discouraged and that, contrary to what previously thought, these patients are characterized by some degree of cognitive difficulties. The term age-related should therefore be applied more correctly to the epileptic syndrome but not to the cognitive outcome. This concept is confirmed by the presence of cognitive as well as behavioral and academic problems, presumably linked to executive functions and processing speed impairment [[35], [36], [37], [38]].

The explorative analyses conducted to examine potential differences in intellectual functioning between the sub-groups of SE did not show any significant results, indicating that the observed intellectual impairment is comparable across the diagnostic subgroups. Nonetheless, the small number of patients within each subgroup warrants caution in interpreting the results. Taken together, this evidence stresses the importance of a detailed neuropsychological assessment immediately at the diagnosis of the epileptic syndrome, allowing the identification of those patients who are at high-risk of experiencing cognitive difficulties and academic underachievement [2,38]. In light of these observations, epilepsy may be considered an epiphenomenon of the underling pathology and not the cause of the intellectual impairment. Hence, the timely and detailed assessment of global intellectual functioning in the three groups of patients identified may be important for the clinician in order to promptly plan the most effective neuropsychological rehabilitation.

Furthermore, our results also confirm that the total duration of epilepsy is related to global intellectual functioning that is to say that increased duration was associated with lower scores in all cognitive indexes. This is in line with previous reports [5,39]. Nevertheless, it is to be noted that in our sample also this parameter, the duration of epilepsy, varied among groups. Specifically, both the UEE and SE group had the longest duration as compared to the ARES group (see Table 2). The statistical significance of this effect was not confirmed within each group, due to the unbalanced and relatively small number of patients. However, at the moment, the literature does not allow to specify the role of factors – such as seizure frequency and chronic pharmacological treatment – in determining the negative impact of the duration of illness. Apparently, the relationship between long duration of illness and more pronounced intellectual impairment seems to contradict some of our previous statements about the determinant role of etiology on this issue. However, the fact that our assessments were not performed at the onset of the epileptic syndrome may implicate that the duration of the disease, as already suggested [40], is itself an indicator of its intrinsic severity.

Interestingly, for what concerns the active duration of the disease, in our study this factor was not found to significantly influence any of the cognitive outcomes. As already mentioned in the Methods, in patients with well controlled seizure the active duration was shorter than the total duration, while in patients with uncontrolled seizures, the active duration corresponded exactly to the total duration. In any case, our data indicate that with exception of the VIQ, the active duration of the disease had a marginally significant effect on both the FSIQ (p = 0.09) and the PIQ (p = 0.08), with longer active duration being associated with lower scores, as already emerged in previous research [24]. The fact that this finding did not reach the full statistical significance may be due to the large number of SE patients in our sample.

The other explanatory factors (i.e., seizure type, ASMs, seizure frequency and age at onset) were not found to be significant predictors of the intellectual functioning. Nonetheless, a near-to-significance result emerged in regards to the seizure type, indicating low FSIQ in children with generalized as compared to children with focal seizures. Similar findings emerged in previous research [10,11]. However, in our study the interaction between this factor and the other clinical variables was not investigated. Despite conflicting evidence can be found in the literature regarding the impact of these factors on cognitive development [12,13], there are studies indicating that seizures frequency and other EEG characteristics can exacerbate the neurocognitive dysfunctions caused by specific etiopathogenic mechanisms [2]. The precise interaction between the etiology of the epileptic syndrome and all these other clinical and instrumental parameters can only be defined by enrolling larger samples of patients.

The present study is not devoid of limitations, most of which depends on its observational nature. However, observational studies may offer critical insights into the outcomes obtained in routine practice provided to a non-selected population, i.e. those patients who would not meet eligibility criteria for participating in methodologically more rigorous research [41]. The so-called “real-world studies”, collecting data gathered from the clinical settings and from populations that are similar to those encountered in clinical practice, are thought to have broader generalizability [42]. Moreover, observational studies allow to obtain data on long-term outcomes.

Additionally, being this study carried out a third-level Scientific Institute, it is possible that even within the population of ARES there may be an over-representation of cases requiring more complex management. However, the finding that patients in this group displayed IQ scores around the normative level seems to suggest that the risk of selection bias may be somewhat limited.

In conclusion, the present findings contribute to an overall picture of intellectual functioning in children and adolescents with epilepsy in relation to the duration and the etiology of the disease. Particularly, our results about the duration of the disease highlight the importance of programming longer follow-up neuropsychological assessments to monitor the slowing of the cognitive development. Furthermore, knowledge about the risks associated with etiology-related factors may help the clinicians to foresee and plan neuropsychological rehabilitation strategies.

## Author contribution statement

Viola Oldrati; Alessandra Bardoni; Claudio Zucca: Conceived and designed the experiments; Analyzed and interpreted the data.

Claudio Zucca; Nicoletta Zanotta; Sara Minghetti: Contributed reagents, materials, analysis tools or data.

Viola Oldrati; Sara Minghetti; Nicoletta Zanotta; Alessandra Bardoni; Claudio Zucca: Wrote the paper.

## Funding statement

This work was supported by the 10.13039/501100003196Ministero della Salute [Ricerca corrente 2022-2023] and 5xMille funds: 5M-2017-23681597 and 5M-2016-23681602.

## Data availability statement

Data will be made available on request.

## Declaration of interest's statement

The authors declare no conflict of interest.
